# An examination of the role of changes in country-level leisure time internet use and computer gaming on adolescent drinking in 33 European countries

**DOI:** 10.1016/j.drugpo.2021.103508

**Published:** 2021-10-29

**Authors:** Rakhi Vashishtha, John Holmes, Amy Pennay, Paul M. Dietze, Michael Livingston

**Affiliations:** a Centre for Alcohol Policy Research, La Trobe University, Melbourne, Australia; b Health Services and Systems Research, Duke-NUS Medical School, Singapore; c School of Health and Related Research, University of Sheffield, Sheffield, UK; d National Drug Research Institute, Curtin University, Australia; e School of Public Health and Preventive Medicine, Monash University, Melbourne, Australia; f Behaviours and Health Risks Program, Burnet Institute, Australia; g Department of Clinical Neurosciences, Karolinska Institutet, Stockholm, Sweden

**Keywords:** Adolescent drinking trends, Adolescent internet use, ESPAD countries

## Abstract

**Introduction:**

Adolescent alcohol consumption has been declining in many high-income countries since the turn of this century. Research investigating the plausible explanations for these declines has been mostly based on individual-level studies, which are largely inconclusive. Changes in leisure time internet use and computer gaming (referred to in this article as ‘computer activities’) have been hypothesised to play a role in declining adolescent alcohol consumption at a country-level. The aim of this study was to examine the association between country-level changes over time in computer activities and adolescent drinking in 33 European countries.

**Methods:**

This is a multi-level repeated cross-national study examining the role of changes over time in country-level and individual-level computer activities on regular drinking. We utilised four waves of the European School Survey Project on Alcohol and Drugs (ESPAD) from 2003, 2007, 2011 and 2015. At an individual-level the primary exposure of interest was daily engagement in computer activities and aggregated means were used to measure country-level daily computer activities in each included country. Data were analysed using three-level hierarchical linear probability methods.

**Results:**

In the fully adjusted model, for between individual effects, we found significant positive association between daily computer activities and regular drinking (*β* = 0.043, p-value <0.001 and 95% CI = 0.033–0.054). However, at a country-level, we did not find any association between within-country changes in daily computer activities and regular drinking (*β* = 0.031, p-value = 0.652 and 95% CI = -0.103–0.164.

**Conclusion:**

Findings from this study suggest that broad cultural shifts towards increased computer-based activities among adolescents has played a little or no role in declining adolescent drinking. Future research should be directed towards examining other high-level cultural changes which may have influenced cross-national reductions in adolescent drinking.

## Introduction

Declines in alcohol consumption among adolescents have been recorded over the last two decades in the majority of high-income countries where regular surveys of adolescent alcohol consumption are conducted (Kraus et al., 2018; Looze et al., 2015; Oldham, Holmes, Whitaker, Fairbrother, & Curtis, 2018; Vashishtha et al., 2020). Nevertheless, the timing and magnitude of these declines has varied substantially, beginning earliest in the USA and Northern Europe, and later in Western Europe and Australasia. In Eastern and Southern European countries, declines have been less consistent, or indeed absent, over time (Looze et al., 2015; Pape, Rossow, & Brunborg, 2018; Vashishtha et al., 2020). Understanding the drivers of this unprecedented decline will provide crucial insights into risk and protective factors for youth drinking and can inform future policy and prevention efforts.

When the declines in drinking were first reported, multiple hypotheses were proposed to explain them (Bhattacharya, 2016; Pape et al., 2018; Pennay, Livingston, & MacLean, 2015). A recent review by our group found 17 empirical papers looking at the drivers of these declines, with parenting factors the most studied, and limited robust evidence examining other factors, such as changes in alcohol policies, increases in immigration, substitution with other illicit substances, weaker economies, exposure to alcohol advertising, and shifts in leisure time activities, namely; involvement in organised sports and parties.

As the research in this area continued to evolve, additional factors have been subsequently empirically tested ranging from additional general parenting factors (Ball, Edwards, Sim, Cook, & Denny, 2020; Kim, Evans, & Hagquist, 2019; Rossow, Pape, & Torgersen, 2020), engagement in healthier lifestyles (Arnett, 2018; Vashishtha, Pennay, Dietze, & Livingston, 2021a), school engagement (Kim et al., 2019; Larm, Åslund, Raninen, & Nilsson, 2018; Raitasalo et al., 2020; Rossow et al., 2020), policy changes (Lintonen, Karlsson, Nevalainen, & Konu, 2013; Mojica-Perez, Callinan, & Livingston, 2020; Vashishtha, Pennay, Dietze, & Livingston, 2021b), perceived access to, and risks of alcohol (Raitasalo et al., 2020), changes to leisure time activities such as in-person socializing with friends (Ball et al., 2020; Chomynová, & Kážmér, 2019; Kim et al., 2019; Raitasalo et al., 2020; Raitasalo, Simonen, Tigerstedt, Mäkelä, & Tapanainen, 2018; Rossow et al., 2020), participation in organised activities (Raitasalo et al., 2020, 2018; Rossow et al., 2020), and changes in population demographics through immigration (Kim et al., 2019). With regards to leisure time specifically, four studies from Europe found a positive association between socializing with friends and the decline in adolescent alcohol consumption (Chomynová & Kážmér, 2019; Kim et al., 2019; Raitasalo et al., 2018; Rossow et al., 2020). However, in contrast, a multinational study from Sweden, Norway and Finland (Raitasalo et al., 2020) and a single country study from New Zealand (Ball et al., 2020) found no significant association between changes in socializing with friends and declining in adolescent drinking. The evidence from these studies is mixed, with some studies showing a positive association for some factors and the decline in some types of alcohol consumption in one country, but no association in another.

A prominent factor that has been proposed as a reason for declining adolescent drinking is a shift in leisure time activities related to increases in internet and computer use. The emergence of widespread internet connectivity in the early 2000s, followed by social media platforms like MySpace, YouTube and Instagram coincide with the timing of the downward trend in adolescent drinking. In addition to this, the widespread use of mobile phones with wireless internet connectivity is likely to have shifted the use of internet from family computers to teenage bedrooms. These online activities, which cross international boundaries, have clearly provided adolescents with an alternative mode of entertainment, communication and socialization over the past two decades (Kraus et al., 2019; Subrahmanyam, Reich, Waechter, & Espinoza, 2008). It has been hypothesised that young people may be spending their leisure time on internet use or gaming rather than going out with friends and drinking (Kraus et al., 2019; Pennay et al., 2015; Room et al., 2019).

Alongside these hypotheses, and potentially part of the reason for mixed results, is that the rise of the internet has provided a new mechanism for alcohol marketing to young people by redefining social norms around drinking and promoting active participation through features such as “stories” and “live feeds” (Carah, Brodmerkel, & Hernandez, 2014; Nicholls, 2012). Recent studies have also shown how internet-based technologies play a central role in coordination of social activities for young people (Hennell, Piacentini, & Limmer, 2020; Supski, Lindsay, & Tanner, 2017). However, empirical studies of the relationship between internet use and drinking or computer gaming and drinking have produced mixed results (Brunborg & Andreas, 2019; Brunborg, Mentzoni, & Frøyland, 2014; Erevik et al., 2019; Ream, Elliott, & Dunlap, 2011; Wenzel, Bakken, Johansson, Götestam, & Øren, 2009). Multiple individual-level studies have identified positive associations between internet use and adolescent drinking (Brunborg & Andreas, 2019; Brunborg, Andreas, & Kvaavik, 2017 Chiao, Yi, & Ksobiech, 2014; Huang et al., 2014; Moreno, Christakis, Egan, Brockman, & Becker, 2012; Mu, Moore, & Lewinn, 2015), while individual-level studies of computer gaming have produced more mixed results (Brunborg et al., 2014; Erevik et al., 2019; Ream et al., 2011; Svensson, 2013; Wenzel et al., 2009). However, these studies have all been either cross-sectional or focussed on particular birth cohorts, and thus provide little insight into how changes in computer activities between generations might relate to changes in drinking.

To date, there have been two individual-level studies that have explicitly examined the role of changes in internet use (Larm, Raninen, Åslund, Svensson, & Nilsson, 2019) and electronic media use (de Looze et al., 2019) and the recent declines in adolescent drinking. Larm et al. examined the role of changes in leisure time internet use and declines in drinking among Swedish adolescents (Larm et al., 2019). The authors found engagement in computer gaming was associated with increased odds of non-drinking between 2008 and 2012, however, time spent on social media/chatting was associated with decreased odds of non-drinking, suggesting complex effects for different types of technological engagement. A multinational study (de Looze et al., 2019) using Health Behaviour in School-aged Children study data examined the role of face-to-face communication and electronic media communication (EMC) separately on declining adolescent drinking. The authors did not find any evidence to support the hypothesis that increased use of EMC was associated with the observed decline in adolescent drinking but found evidence that declining face-to-face communication was associated with the observed decline in adolescent drinking.

Most of the previous research on digital technology use and adolescent alcohol consumption has focussed on individual-level associations. More recently, the importance of conducting cross-national country-level analyses has been emphasised given the international nature of declines in adolescent drinking (Vashishtha & Livingston, 2020). In this study, we hypothesized that broad cultural shifts in technology use among adolescents have influenced the social opportunities and activities engaged in among young people. Thus, the decline in youth drinking may have been driven by both individual-level engagement in computer activities but also by broader sociocultural shifts across the population of young people. It is important to note that individual-level associations may not provide clear insights into country-level changes. The fact that individual-level associations can be in the opposite direction to country-level associations, known as Simpson’s paradox (Bracey, 2005), highlights the importance of assessing country-level associations when investigating potential explanations for changes that occur across countries.

In the only longitudinal multi-country study to take a population approach, Halkjelsvik, Brunborg, and Bye (2020) used European School Project on Alcohol and Drugs (ESPAD) data to examine the association between country-level changes in computer gaming and heavy episodic drinking, which is having five or more drinks on a single occasion, in 35 countries between 1995 and 2015, but did not find any association. While this study focussed on computer gaming and its role in declining heavy episodic drinking, the authors did not adjust for or include any other form of internet activity or examine drinking participation more broadly. Focussing only on changes in computer gaming risks missing the very substantial changes in the use of the internet for other activities such as chatting, sharing photos, and social media, for example.

In this study, we attempted to address the limitations of previous research by using a multi-country design in which we examined simultaneous changes in both computer gaming and internet use at a country-level over time. This study uses repeat cross-sectional survey data in a multi-level analysis of individuals (Level 1) and country-years (Level 2). The primary aim of this study was to extend the work of Halkjelsvik, Brun-borg, and Bye (2020) by examining the country-level associations between changes over time in leisure time computer activities and the declines in adolescent regular alcohol consumption observed in the ES-PAD data. The secondary aim was to examine if other individual-level leisure time activities confounded this association.

## Methods

### Data source

The ESPAD survey is designed to collect comparable data across European countries on substance use behaviours among 15–16-year-olds. The survey has been conducted every fourth year starting from 1995. We used data from four ESPAD surveys spanning 2003 to 2015 because internet use data was not collected before 2003. A total of 35 European countries participated in each wave from 2003 to 2015. In the present study, we included only countries which provided data on regular drinking and leisure time internet use or computer gaming for at least three time points between 2003 and 2015. Based on these eligibility criteria, 33 out of 35 countries were included. This includes countries with varying adolescent drinking trends, allowing for stronger inference about the role of leisure time internet use or computer gaming in driving changes in adolescent drinking.

Sampling was carried out using a cluster sampling design in each participating country. Stratification by region and municipality was used to randomly select schools. The final unit for sampling was class, which was selected using a multi-stage sampling procedure. Student participation was voluntary and anonymity was ensured. Weights were provided for countries where non-proportionate stratification was used for sampling and these sampling weights were applied to account for sampling design where country samples were not representative. Data collection was conducted via self-administered questionnaire. More information on survey methodology can be found elsewhere (ESPAD Report 2015: Results from the European school survey project on alcohol & other drugs, 2016; Hibell & Bjarnason, 1997; Hibell et al., 2000, 2009; Hibell et al., 2012). The sample sizes and response rates for all countries are provided in Supplementary Table 1.

### Measures

### Dependent variable

The main outcome of interest was regular drinking, primarily because we expect that the mechanism linking computer activities (a daily activity) and youth drinking would be strongest for more frequent alcohol use. Previous work (Halkjelsvik, Brunborg, & Bye, 2020) examined heavy episodic drinking and computer gaming, finding no significant relationship. We have included a heavy episodic drinking model presented in Supplementary Table 5 for completeness. We conceptualised regular drinking as at least three drinking occasions per month. It was measured by asking participants “How many occasions have you consumed any alcoholic beverage during the last 30 days”. Response categories were ‘0’, ‘1–2’, ‘3–5’, ‘6–9’, ‘10–19’, ‘20–39’ and ‘40 or more’. To form a dichotomous categorical variable for the regular drinking measure, all responses of ≥ 3–5 times were combined and assigned a value of “1”, and “0” where less than 3–5 drinking occasions were reported in the last 30 days. Heavy episodic drinking was measured by asking participants “How many times (if any) have you had five or more drinks on one occasion during last 30 days? Response categories were ‘0’, ‘1’, ‘2’, ‘3–5’, ‘6–9’, ‘10+’. To form a dichotomous categorical variable for the heavy episodic drinking measure, all responses of > = 1 time were combined and assigned a value of “1”, and “0” where 0 heavy episodic drinking occasions were reported in the last 30 days.

### Independent variables

To measure leisure time internet activities participants were asked “how often do you use the internet for leisure activities (chats, music, games, social networks, videos etc.)?” Similarly, participants were asked “how often do you play computer games?” The response options for both questions were “Never”, “A few times a year”, “Once or twice a month”, “At least once a week”, and “Almost every day”. Considering the problems that may arise for participants in an attempt to disentangle their internet use, computer gaming and internet-based computer gaming, we combined the use of either internet or computer games into one main exposure variable of interest referred to as computer activities. The two types of activities were combined (hereafter computer activities) and dichotomized to measure near-daily use of one or both. If participants responded ‘almost everyday use’ to either computer gaming or internet use (or both) they were classified as ‘almost everyday use’ on our combined computer activities variable. Leisure time internet use and computer gaming were also examined separately in sensitivity analyses (see Analysis section).

### Other variables

One of the important predictors included in the study was internet penetration in each country during every survey year i.e. the prevalence of overall internet use in the population. We referred to this measure as “societal internet use”. The prevalence of societal internet use across the entire population aimed to capture any shifts due to increases in internet use on adolescent alcohol use. Data were derived from World Bank Internet data repository (Bank, 2019). World Bank Data on internet use is sourced through the International Telecommunication Union which collaborates with national agencies and combines subscription, access and use data on telecommunication services. We also adjusted for other leisure time activities, namely: engagement in sports, reading, going out, and hobbies. These other activities may be substituted by computer activities and also drinking, and thus may act as confounders (Hagell, Peck, & Zarrett, 2012; Wight, Price, Bianchi, & Hunt, 2009). These other leisure activities were dichotomised based on frequency in line with measures of drinking and computer activities i.e. “regular” and “less regular”. Gender was also added as another potential confounder because the use of the internet, computer gaming and drinking vary significantly by gender (de Looze et al., 2019; Larm et al., 2019). Perceived parental affluence was also added as a proxy covariate for socioeconomic status.

### Analysis

We utilised mixed effect linear probability modeling techniques involving a three-level hierarchical multi-level model (MLMs). Due to convergence issues, we used linear rather than logistic models for our binary outcome. This means the positive value for our key parameter of interest, i.e. daily use of computer activities, describes increase in probability of regular drinking (Chatla & Shmueli, 2016).

For these models, participants (level 1) were nested within country-years (level 2), which were further nested within countries (level 3). As our primary aim was to examine the effect of computer-based activities on drinking at an aggregate or population level over time, we used the approach suggested by Fairbrother (2011) for analysing repeated waves of cross-sectional survey data and producing unbiased estimates of effects both over time within countries and consistent differences between countries. Fairbrother et al.’s approach focusses on time-varying country level variables, which for computer activities we derived from the individual survey responses. We first computed the mean prevalence of computer activities for each country across years, which can be used to estimate the effect of between country differences in computer-based activities on adolescent drinking. Subsequently, we calculated a second variable, by subtracting the country mean from the mean for each country-year, to ensure interpretable country-year (i.e. within) and country (i.e. between) estimates. Finally, to estimate easily interpretable individual-level effects, corresponding country-year means were then subtracted from each individual-level participant response to derive a new individual measure adjusted for all population effects.

To adjust for societal internet use prevalence, similar to our primary exposure of interest (computer activities), we derived average societal internet use prevalence over the four survey years for each country. In addition, to measure the within country effects in societal internet use the country mean prevalence was subtracted from mean prevalence in each survey year.

For the main analysis, 12 country-years were not included as those were missing on either computer activities, drinking or both, namely: Croatia and Malta were missing on computer activities (mainly internet use) in 2003, Faroe Island was missing on drinking in 2007, and Portugal in 2015 and France in 2003 on family affluence. Austria in 2011, Ireland, Moldova, and Montenegro in 2003, and Germany, Russia and UK in 2015, were missing on both alcohol and computer activities. To examine the effects of individual-level as well as aggregated-level variables, a total of three models are presented. In model 1, we examined the effect of survey years on regular drinking. In model 2, individual-level covariates were added. In model 3, combined individual-level as well as aggregated-level covariates were added. To account for clustering at class and school levels, for which data are unavailable, and the inherent heteroskedasticity resulting from the chosen modeling technique, we calculated cluster-robust standard errors adjusted at the level of countries.

We also ran five sensitivity analyses, that 1) examined the association between daily internet use only and regular drinking, 2) examined the association between daily computer gaming only and regular drinking, 3) examined the association between daily computer activities for boys and girls separately, 4) heavy episodic drinking as an outcome of interest and 5) examined the association between regular drinking and population-level computer based activities without societal-level internet use. The first two sensitivity analyses helped us examine whether there were differential effects of internet use and computer gaming on adolescent drinking. The third sensitivity analysis was carried out to examine whether the associations differed between genders because there is evidence from previous studies (de Looze et al., 2019; Larm et al., 2019) that the association between internet or computer gaming and drinking differ between two gender categories. The fourth sensitivity analysis was conducted to examine whether changes in individual-level or population-level computer activities were associated with changes in heavy episodic drinking among 15-year-olds. The final sensitivity analysis was carried out to examine if adjusting for societal-level internet use could bias the results due to its relationship with self-reported internet use by young people.

## Results

**Figure 1:**
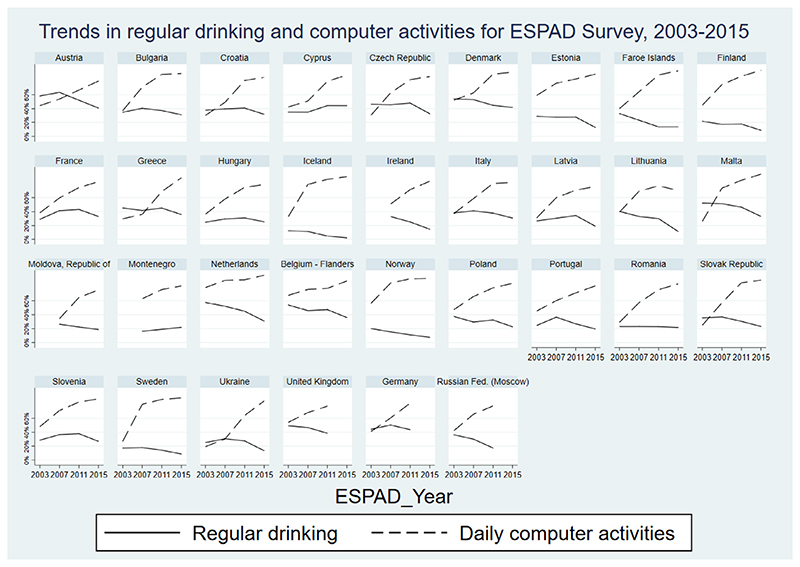
Trends in regular drinking and computer activities for ESPAD survey, 2003–2015.

Based on our eligibility criteria, the total sample for the main analysis consisted of 361,998 participants from 33 countries and 120 country-years. Fig. 1 illustrates the trends in regular drinking and computer activities between 2003 and 2015 for each included country. The weighted prevalence rates for regular drinking declined for almost all countries except Montenegro and Cyprus (Supplementary Table 2). Broadly speaking, there was an increase in the proportion of those who either used the internet daily or played computer games daily between 2003 and 2015 across all countries (Supplementary Table 3). However, for thirteen countries (Cyprus, Denmark, France, Greece, Hungary, Lithuania, Malta, Netherlands, Poland, Portugal, Romania, Slovenia, and Sweden), the prevalence of daily computer activities decreased between 2011 and 2015, driven by reductions in gaming.

Model results are presented in Table 1. In the simple Model 1, across our entire sample we found an overall decline in regular drinking between 2003 and 2015. Model 2 showed that, at an individual-level after adjusting for other individual-level covariates, computer activities were found to be significantly positively associated with regular drinking (*β* = 0.043, p-value <0.001 and 95% CI = −0.033-0.054). In Model 3, we included our main exposure of interest – population-level computer activities – and did not find any of these variables to be significantly associated with regular drinking. In other words, we found between country differences and within country changes not to be significantly associated with drinking. However, changes within population-level societal internet prevalence was not found to be associated with regular drinking (*β* = −0.164, p-value = 0.065 and 95% CI = −0.338-0.010).

As noted earlier, we conducted five sensitivity analyses to examine: 1) internet use alone, 2) computer gaming alone, 3) gender specific relationships for combined computer activities, 4) heavy episodic drinking as an outcome of interest, and 5) without adjusting for societal-level internet prevalence. All five sensitivity analyses produced the same null results on our key country-level variables as the main analysis presented here. The results from these sensitivity analyses are provided in supplementary Tables 4 for sensitivity analyses 1–3 (internet use alone, computer gaming alone and gender-specific associations) and supplementary Table 5 for sensitivity analysis 4 and 5 (heavy drinking as an outcome of interest and without adjusting for societal-level internet use, respectively).

## Discussion

In this study, we examined the association between individual-level and country-level daily computer activities and regular drinking among adolescents from 33 European countries. In our fully adjusted model, we found that, for between individual effects, there was a significant positive association between regular drinking and daily computer activities, suggesting that 15-year-olds who engage in daily computer activities tend to drink more than those who don’t. However, at the country-level, we did not find any evidence of an association between within country changes in daily computer activities and regular drinking. Similarly, there was no evidence of a association between societal internet use prevalence and regular drinking prevalence among adolescents.

The purpose of this study was to examine if broad cultural shifts in computer-based activities by adolescents have influenced declining adolescent drinking, which has been observed across multiple high-income countries. However, based on the findings, we found no evidence to suggest that these shifts have contributed to the decline in adolescent drinking. Surprisingly, our computer activities measure declined in 13 countries between 2011 and 2015. This was driven by self-reported declines in gaming, which may relate to decreased popularity of gaming but may also be influenced by other factors such as shifts from PC/console gaming to online/phone-based gaming, which may be difficult for respondents to distinguish from broader internet use.

Our findings align with two recent multi-country studies which found no link between computer gaming (Halkjelsvik, Brunborg, & Bye, 2020) or electronic media communication (de Looze et al., 2019) and the decline in youth drinking. Therefore, even though the timing of internet penetration and declining adolescent drinking overlap, and the individual-level study by Larm et al. (2019) identified some evidence of an association (i.e. positive association for computer gaming and non-drinking and negative association for internet use and non-drinking)in Sweden, changes over time in these leisure-based computer activities do not seem to be associated with the decline in adolescent drinking across 33 European countries.

A major limitation of quantitative studies on technology and drinking is challenges related to measuring internet use and other forms of gaming. There are many forms of gaming, including PC-based gaming, phone gaming, gaming consoles, each of which might be internet based or offline. Some might involve playing against strangers, some with friends, some alone. Similarly, with internet use activities, it can be difficult for individuals to disaggregate between news reading, social media use and online communication, particularly when internet use occurs in highly dynamic ways across static (PCs), mobile (laptops) and highly mobile (smartphone) platforms. Svensson and Johnson (2020) have shown in a recent study the importance of disaggregating internet use activities, with not all internet activities having the same association with adolescent drinking. For example, online sociality and self-presentation were found to be positively associated with drinking, however, reading news online and playing games were found to be negatively associated. Even single activities (e.g. social media use) cover a wide range of behaviours (e.g. online conversation, content sharing, news updates) that differ markedly across platforms (e.g. Facebook has greater family surveillance, Instagram more links between friends (Törrönen, Roumeliotis, Samuelsson, Room, & Kraus, 2020)). Therefore, how leisure time computer activities are measured is likely to be important given the nuances of engagement with different technologies and leisure time pursuits. Crude survey measures may fail to capture the complexity of digital media use by young people. The continued use of internet via mobile devices also makes the measurement of social media engagement through survey measures such as “hours spent on social media” difficult. More qualitative work may help us understand how internet and computer-based activities shape and are shaped by adolescent drinking practices.

Our study is thus limited by the measures of computer activities available consistently in the ESPAD survey. This means we could not measure the dose-related effect of internet use or computer gaming (e.g. number of hours per day), or the effect of various types of leisure-based internet activities on adolescent drinking. Our sensitivity analyses, where we separated the two activities, identified no evidence of an association for gaming and internet use independently, in drinking changes in the population. Our reliance on self-reported measures of drinking and internet use or gaming may also introduce bias to the study. However, self-reported data collected from school surveys has been found to be adequately reliable (Smit, Zwart, Spruit, Monshouwer, & Ameijden, 2002).

Despite the limitations in measuring computer-based activities, the main strength of this study is that it utilizes the most comprehensive available data on internet use and gaming and alcohol consumption over twelve years and 33 countries. Based on the results from this study, it appears that the broad cross-national declines in adolescent drinking might be driven by country-level changes over time in factors other than leisure-based digital technology use. To understand such associations, a similar analytical approach used in this study could be extended to other cultural changes such as changes related to parenting, social norms and attitudes around alcohol, perceived risks of alcohol consumption, and perceived access to alcohol, which to date have mostly been investigated at the country level (Ball et al., 2020; Raitasalo et al., 2020; Rossow et al., 2020). Furthermore, future research in this area could be directed towards incorporating various hypothesized factors together in a more complex analytical model to examine the effect of change over time in different plausible country-level factors on declining adolescent drinking. However, it may remain difficult to identify a single dominant cause of the decline in adolescent drinking due to the lack of a multi-national survey where multiple plausible factors of decline can be studied together.

In conclusion, the findings from this study suggest that broad cultural shifts towards increased computer-based activities among adolescents has played a little or no role in declining adolescent drinking. Future research should be directed towards examining other high-level cultural changes which may have influenced cross-national reductions in adolescent drinking.

## Supplementary Material

Supplementary Material

## Figures and Tables

**Table 1:** Results from MLM models showing association between regular drinking, daily computer activities and other individual-level level explanatory variables.

		Model 1 Without individual or contextual daily computer activities			Model 2 With individual-level daily computer activities			Model 3 With individual-level and contextual-level daily computer activities		
		*β*	p-value	95% CI	B	p-value	95% CI	*β*	p-value	95% CI
	Intercept	0.347	<0.001	0.304–0.388	0.339	<0.001	0.294–0.384	0.480	0.002	0.175-0.785
**Survey years**	2003	Reference			Reference			Reference		
	2007	0.003	0.819	−0.019-0.024	−0.010	0.351	−0.030- 0.011	−0.034	0.075	−0.071-0.003
	2011	−0.026	0.072	−0.054-0.002	−0.032	0.014	−0.057- 0.006	−0.074	0.009	−0.128- −0.019
	2015	−0.106	<0.001	−0.136- −0.075	−0.088	<0.001	−0.118- −0.059	−0.144	<0.001	−0.215 - −0.073
**Individual-level variables**	Between individual effect of daily computer activities				0.043	<0.001	0.033-0.054	0.043	<0.001	0.033- 0.054
**Gender**	Male				Reference			Reference		
	Female				−0.068	<0.001	−0.088- −0.048	-0.068	<0.001	−0.088- −0.048
**Family affluence**	Better off				Reference			Reference		
	About the same				−0.012	0.002	−0.019- −0.004	−0.012	0.002	−0.019- −0.004
	Less well off				0.005	0.398	−0.007-0.018	0.005	0.397	−0.007-0.018
	Less than once a week				Reference			Reference		
**Leisure time activities**	Leisure time sports (Once a week)				−0.007	0.107	−0.016-0.002	−0.007	0.107	−0.016-0.002
	Leisure time reading books (Once a week)				−0.053	<0.001	−0.063- −0.043	−0.053	<0.001	−0.063- −0.043
	Leisure time going out (Once a week)				0.263	<0.001	0.233-0.293	0.263	<0.001	0.233-0.293
	Leisure time hobbies (Once a week)				−0.021	<0.001	−0.028- −0.014	−0.021	<0.001	−0.028- −0.014
**Contextual level**	Between country differences in daily computer activities							−0.271	0.335	−0.840-0.298
**variables**	Within- country changes (from country mean) in daily computer activities							0.031	0.652	−0.103-0.164
	Between country differences in societal internet prevalence							0.128	0.658	−0.160-0.416
	Change within countries from country mean in societal internet prevalence							−0.164	0.065	−0.338-0.010
**Random effects**	Country level variance	0.012		0.008-0.017	0.013		0.008-0.019	0.012		0.008-0.019
	Country-year level variance	0.002		0.001-0.003	0.002		0.001-0.002	0.002		0.001-0.002
