# Supplementary material for: An examination of the role of changes in country-level leisure time internet use and computer gaming on adolescent drinking in 33 European countries
Source: Int J Drug Policy. Author manuscript; Available in PMC 2023 Aug 11. (PMC7614941; doi:10.1016/j.drugpo.2021.103508)
Supplement: Supplementary Material [file EMS184780-supplement-Supplementary_Material.docx]

**Supplementary tables**

Supplementary Table 1 – Sample size and class (school) participation rate for ESPAD surveys 2003–2015 by country.

|  | Sample size | | | | Participation rates | | | |
| --- | --- | --- | --- | --- | --- | --- | --- | --- |
|  | **2003** | **2007** | **2011** | **2015** | **2003** | **2007** | **2011** | **2015** |
| Austria | 2402 | 2571 | NA | 3684 | 90 | 91 |  | 90 |
| Belgium | 1291 | 1889 | 1798 | 1771 | 81^b, d^ | 95 | 95 | 94 |
| Bulgaria | 2666 | 2353 | 2217 | 2922 | 85 | 86 | 82 | 84 |
| Croatia | 2852 | 3008 | 3002 | 2558 | 88 | 89 | 89 | 89 |
| Cyprus | 2142 | 6340 | 4243 | 2098 | 88 | NA | 83 | NA |
| Czech Republic | 3149 | 3901 | 3913 | 2738 | 95 | 89 | 89 | 83 |
| Denmark | 2504 | 877 | 2181 | 1670 | 89 | 87 | 89 | 88 |
| Estonia | 2431 | 2372 | 2460 | 2452 | 86 | 79 | 82^e^ | 83 |
| Faroe Islands | 582 | 552 | 557 | 511 | 86 | 82 | 87 | 92 |
| Finland | 3219 | 4988 | 3744 | 4049 | 91 | 91 | 90 | 89 |
| France | 2277 | 2916 | 2572 | 2714 | 91 | 90 | 87 | 87 |
| Germany ( excl.Bavaria) | 810 | 814 | 724 | 862 | NA | NA | NA | NA |
| Greece | 1891 | 3060 | 5919 | 3202 | 83 | 91 | 90 | 92 |
| Hungary | 3109 | 2817 | 3063 | 2735 | 82 | 89 | 86 | 85 |
| Ireland | NA | 2221 | 2207 | 1470 | 96 | 94 | 94 | 86 |
| Italy | 4818 | 9981 | 4837 | 4059 | 98 | 88 | 86 | 88 |
| Latvia | 2816 | 2275 | 2622 | 1119 | 84^b^ | 83 | 85 | 85 |
| Lithuania | 5028 | 2411 | 2476 | 2573 | 88 | 86 | 89 | 88 |
| Malta | 3443 | 3668 | 3377 | 3326 | 83 | 84 | 78 | 83 |
| Moldova | NA | 3176 | 2162 | 2586 | NA | NA | 83 | 87 |
| Montenegro | NA | 5823 | 3387 | 3844 | NA | NA | 91 | 87 |
| Netherlands | 2068 | 2091 | 2044 | 1684 | 93^b^ | 93 | NA | 90 |
| Norway | 3745 | 3482 | 2938 | 2584 | 87^c^ | 89 | 88 | 93 |
| Poland | 5842 | 2120 | 5934 | 11822 | 85 | 84 | 82 | 83 |
| Portugal | 2919 | 3141 | 1965 | 3456 | 96 | 96 | 91 | 93 |
| Romania | 4371 | 2289 | 2770 | 3500 | 84 | 84 | 79 | 84 |
| Slovak Republic | 2122 | 2468 | 2009 | 2208 | 87 | 89 | 82 | 89 |
| Slovenia | 2758 | 3085 | 3186 | 3484 | 88 | 86 | 89 | 88 |
| Sweden | 3212 | 3179 | 2569 | 2551 | 87 | 84 | 85 | 86 |
| Ukraine | 4173 | 2447 | 2210 | 2350 | 83 | 82 | 83 | 80 |
| United Kingdom | 2003 | 2179 | 1712 | NA | 84^b^ | 84 | 81 | NA |

^a^ Participating students in participating classes

^b^ Calculated on all students in participating classes

^c^An estimate not based on classrooms reports. It shows the proportion of participating students out of all 1987 born students in the country and not the number of students in participating classes.

^d^93% in Flemish and 74% in French speaking schools

^e^ calculated in a different way than other countries

NA = not available

Supplementary Table 2 – Weighted prevalence of regular drinkers, ESPAD Survey, 2003-2015

|  | 2003 | 2007 | 2011 | 2015 | Country Mean |
| --- | --- | --- | --- | --- | --- |
| Austria | 58.1 (56.1-60.1) | 63.3 (61.4-65.2) | NA | 40.2 (38.6-41.9) | 52.0 (50.9-53.0) |
| Belgium | 54.0 (51.2-56.7) | 46.0 (43.7-48.2) | 47.4 (45.0-49.9) | 35.8 (33.6-38.1) | 45.1 (43.9-46.3) |
| Bulgaria | 34.7 (32.9-36.6) | 40.4 (38.4-42.4) | 37.2 (35.2-39.2) | 31.2 (29.5-32.9) | 35.6 (34.6-36.5) |
| Croatia | 45.4 (42.1-48.7) | 39.5 (37.8-41.3) | 40.8 (39.0-42.6) | 31.8 (30.0-33.7) | 37.6 (36.6-38.7) |
| Cyprus | 35.0 (33.0-37.1) | 34.9 (33.7-36.1) | 44.3 (42.8-45.8) | 44.2 (42.1-46.4) | 38.9 (38.0-39.7) |
| Czech Republic | 46.2 (44.4-48.0) | 45.6 (44.0-47.1) | 47.9 (46.4-49.5) | 32.1 (30.3-34.0) | 43.6 (42.7-44.4) |
| Denmark | 53.9 (51.9-55.9) | 52.9 (49.6-56.2) | 44.7 (42.6-46.9) | 41.6 (39.2-44.0) | 48.1 (46.9-49.2) |
| Estonia | 28.9 (27.1-30.8) | 27.7 (25.9-29.5) | 27.8 (26.1-29.6) | 12.9 (11.6-14.3) | 24.2 (23.3-25.1) |
| Faroe Islands | 32.9 (29.1-36.8) | NA | 13.7 (11.1-16.9) | 13.8 (11.1-17.1) | 20.3 (18.3-22.3) |
| Finland | 21.8 (20.4-23.2) | 17.3 (16.3-18.4) | 17.8 (16.6-19.1) | 8.9 (8.0-9.8) | 16.1 (15.5-16.7) |
| France | 29.0 (27.2-31.0) | 40.1 (38.3-41.9) | 42.4 (40.1-44.7) | 29.8 (27.8-31.9) | 35.5 (34.6-36.5) |
| Germany (excl.Bavaria) | 43.8 (42.1-45.5) | 50.4 (48.9-51.9) | 41.5 (38.8-44.2) | NA | 47.2 (46.1-48.2) |
| Greece | 45.3 (43.0-47.6) | 41.7 (39.9-43.4) | 43.1 (41.5-44.7) | 35.1 (33.3-36.9) | 41.2 (40.3-42.0) |
| Hungary | 24.7 (23.2-26.4) | 29.3 (27.6-31.1) | 30.3 (28.5-32.2) | 25.3 (23.7-27.0) | 27.5 (26.6-28.3) |
| Iceland | 12.5 (11.4-13.7) | 11.5 (10.5-12.6) | 5.0 (4.3-5.7) | 2.5 (1.9-3.1) | 7.6 (7.0-8.1) |
| Ireland | NA | 32.9 (31.0-34.9) | 25.2 (23.4-27.0) | 14.8 (13.0-16.7) | 25.4 (24.2-26.5) |
| Italy | 38.2 (36.8-39.6) | 41.1 (40.2-42.1) | 37.9 (36.6-39.3) | 30.8 (29.4-32.3) | 38.1 (37.4-38.7) |
| Latvia | 26.7 (25.0-28.4) | 30.6 (28.7-32.5) | 33.8 (32.0-35.7) | 18.2 (15.3-21.5) | 28.7 (27.7-29.7) |
| Lithuania | 40.3 (39.0-41.7) | 33.1 (31.2-35.0) | 30.0 (28.2-31.8) | 11.5 (10.3-12.8) | 31.0 (30.1-31.8) |
| Malta | 52.3 (50.6-54) | 51.5 (49.8-53.1) | 46.4 (44.8-48.1) | 33.1 (31.6-34.8) | 43.9 (42.9-44.9) |
| Moldova | NA | 26.3 (24.8-27.9) | 22.3 (20.6-24.1) | 18.7 (17.2-20.2) | 22.7 (21.7-23.6) |
| Montenegro | NA | 16.2 (15.3-17.2) | 18.9 (17.7-20.3) | 21.9 (20.6-23.2) | 18.6 (17.8-19.2) |
| Netherlands | 54.5 (51.7-57.2) | 51.0 (48.6-53.5) | 44.9 (42.7-47.2) | 30.2 (28-32.4) | 45.8 (44.6-46.9) |
| Norway | 20.1 (18.8-21.4) | 15.3 (14.1-16.7) | 11.2 (10.0-12.5) | 6.6 (5.6-7.8) | 13.9 (13.3-14.5) |
| Poland | 34.7 (33.2-36.2) | 29.5 (27.6-31.5) | 31.5 (30.0-33.2) | 22.4 (21.3-23.4) | 29.6 (29.0-30.1) |
| Portugal | 24.6 (23.0-26.2) | 36.4 (34.7-38.2) | 26.8 (24.9-28.9) | 19.5 (18.2-20.9) | 26.7 (25.8-27.5) |
| Romania | 23.6 (22.1-25.1) | 24.8 (22.9-26.9) | 23.8 (22.1-25.7) | 21.6 (20.3-23) | 23.3 (22.5-24.0) |
| Russia | 36.4 (34.2-38.6) | 30.1 (28.1-32.2) | 17.4 (15.7-19.3) | NA | 27.3 (26.1-28.5) |
| Slovak Republic | 35.4 (33.4-37.5) | 36.8 (34.9-38.7) | 30.4 (28.4-32.5) | 23.1 (21.4-24.9) | 31.5 (30.5-32.5) |
| Slovenia | 28.7 (27.0-30.4) | 36.6 (34.9-38.3) | 37.9 (36.3-39.6) | 26.9 (25.4-28.4) | 32.5 (31.6-33.3) |
| Sweden | 17.3 (16.0-18.6) | 18.0 (16.7-19.4) | 14.2 (12.9-15.6) | 8.8 (7.7-9.9) | 14.8 (14.1-15.5) |
| Ukraine | 26.5 (25.0-28.0) | 30.5 (28.7-32.4) | 27.6 (25.7-29.5) | 15.4 (13.8-17.1) | 25.1 (24.3-26.0) |
| United Kingdom | 49.3 (47.1-51.6) | 46.8 (44.6-48.9) | 38.8 (36.5-41.2) | NA | 45.3 (44.0-46.6) |

Supplementary Table 3 Weighted prevalence of daily computer activities, ESPAD Survey, 2003-2015

|  | 2003 | 2007 | 2011 | 2015 | Country Mean |
| --- | --- | --- | --- | --- | --- |
| Austria | 44.8 (42.7-46.8) | 54.4 (52.4-56.3) | NA | 80.2 (78.8-81.5) | 62.8 (61.7-63.8) |
| Belgium | 67.8 (65.2-70.3) | 77.0 (75.0-78.8) | 79.0 (76.9-81.0) | 89.2 (87.6-90.5) | 79.0 (78.0-80.0) |
| Bulgaria | 37.6 (35.8-39.5) | 71.5 (69.6-73.3) | 89.5 (88.2-90.7) | 90.9 (89.7-91.9) | 72.2 (71.3-73.1) |
| Croatia | NA | 49.3 (47.5-51.1) | 80.5 (79.1-81.9) | 85.1 (83.7-86.5) | 71.0 (70.0-72.0) |
| Cyprus | 42.5 (40.4-44.6) | 51.2 (49.9-52.4) | 82.1 (79.3-81.7) | 88.6 (87.2-89.9) | 63.7 (62.9-64.5) |
| Czech Republic | 31.0 (29.4-32.7) | 63.2 (61.6-64.7) | 82.1 (80.8-83.3) | 86.4 (85.0-87.7) | 66.1 (65.2-66.9) |
| Denmark | 52.4 (50.4-54.3) | 63.1 (59.8-66.2) | 89.9 (88.5-91.1) | 94.8 (93.6-95.8) | 74.7 (73.7-75.7) |
| Estonia | 59.4 (57.4-61.3) | 76.9 (75.1-78.6) | 83.6 (82.1-85.0) | 89.7 (88.5-90.9) | 77.5 (76.6-78.3) |
| Faroe Islands | 40.6 (36.6-44.7) | NA | 88.8 (85.9-91.2) | 94.6 (92.2-96.3) | 73.6 (71.7-75.4) |
| Finland | 45.0 (43.3-46.7) | 74.6 (73.4-75.8) | 86.6 (85.5-87.7) | 95.0 (94.3-95.7) | 76.6 (76.0-77.3) |
| France | 39.0 (36.9-41.0) | 61.3 (59.4-63.1) | 75.6 (73.5-77.6) | 84.7 (83.1-86.3) | 66.1 (65.1-67.1) |
| Germany ( exc.Bavaria) | 41.4 (39.7-43.2) | 61.0 (59.5-62.5) | 83.1 (81.0-85.0) | NA | 58.0 (56.9-59.1) |
| Greece | 29.7 (27.7-31.9) | 36.3 (34.6-38.0) | 70.9 (69.4-72.3) | 88.2 (86.9-89.4) | 62.0 (61.1-62.9) |
| Hungary | 36.5 (34.7-38.3) | 58.2 (56.3-60.1) | 75.5 (73.7-77.2) | 80.6 (79.0-82.0) | 63.2 (62.2-64.1) |
| Iceland | 65.8 (63.5-68.1) | 79.5 (78.2-80.8) | 86.8 (85.6-87.9) | 91.4 (90.3-92.4) | 82.5 (81.8-83.2) |
| Ireland | NA | 51.5 (49.4-53.6) | 72.4 (70.5-74.3) | 87.1 (85.2-88.8) | 68.3 (67.1-69.5) |
| Italy | 37.6 (36.2-39.0) | 59.0 (58.0-60.0) | 80.6 (79.5-81.7) | 82.3 (81.1-83.5) | 63.0 (62.4-63.6) |
| Latvia | 31.2 (29.5-33.0) | 60.4 (58.4-62.5) | 70.5 (68.6-72.3) | 75.6 (71.8-79.1) | 56.1 (55.0-57.3) |
| Lithuania | 39.3 (37.9-40.6) | 69.7 (67.8-71.6) | 77.4 (75.7-79.0) | 71.1 (69.3-72.8) | 59.2 (58.3-60.1) |
| Malta | NA | 74.0 (72.5-75.4) | 85.3 (84.0-86.4) | 94.3 (93.4-95.0) | 84.3 (83.6-85.0) |
| Moldova | NA | 34.7 (33.0-36.4) | 65.3 (63.3-67.4) | 76.2 (74.5-77.8) | 56.9 (55.8-58.0) |
| Montenegro | NA | 63.4 (62.2-64.7) | 77.4 (75.9-78.8) | 82.3 (81.0-83.4) | 72.7 (71.9-73.5) |
| Netherlands | 79.7 (77.5-81.7) | 88.9 (87.2-90.4) | 89.8 (88.3-91.1) | 96.6 (95.6-97.4) | 88.4 (87.6-89.2) |
| Norway | 57.9 (56.2-59.5) | 86.5 (85.2-87.7) | 92.9 (91.8-93.9) | 95.9 (94.9-96.8) | 81.5 (80.8-82.2) |
| Poland | 41.8 (40.2-43.4) | 66.5 (64.5-68.5) | 78.4 (76.9-79.8) | 84.7 (83.7-85.6) | 66.2 (65.4-67.0) |
| Portugal | 45.6 (43.7-47.5) | 60.6 (58.8-62.3) | 71.8 (69.7-73.8) | 81.5 (80.1-82.8) | 65.2 (64.3-66.0) |
| Romania | 31.6 (29.9-33.3) | 60.0 (57.7-62.2) | 77.6 (75.9-79.3) | 85.2 (83.9-86.3) | 61.3 (60.4-62.3) |
| Russia | 42.9 (40.6-45.2) | 66.4 (64.3-68.5) | 79.0 (77.0-80.8) | NA | 60.8 (59.2-62.3) |
| Slovak Republic | 25.0 (23.2-26.9) | 57.4 (55.4-59.3) | 86.1 (84.5-87.6) | 89.8 (88.4-91.0) | 64.2 (63.2-65.2) |
| Slovenia | 48.7 (46.8-50.6) | 71.4 (69.7-73.0) | 84.1 (82.8-85.4) | 88.3 (87.1-89.3) | 74.5 (73.7-75.3) |
| Sweden | NA | 80.7 (79.2-82.0) | 87.8 (86.5-89.1) | 90.3 (89.1-91.4) | 87.3 (86.6-88.0) |
| Ukraine | 20.5 (19.2-21.9) | 31.7 (29.8-33.6) | 64.4 (62.4-66.4) | 85.5 (83.9-87.0) | 45.8 (44.8-46.8) |
| United Kingdom | 54.5 (52.3-56.7) | 68.4 (66.4-70.3) | 77.8 (75.7-79.8) | 80.2 (78.8-81.5) | 66.4 (65.1-67.6) |

Supplementary Table 4 – Results from four sensitivity analyses

|  |  | Model S1 Including both individual and contextual daily leisure time internet use alone | | | Model S2 Including both individual and contextual daily leisure time computer gaming alone | | | Model S3 With both individual and contextual daily leisure time combined computer activities for boys only | | | Model S4 With both individual and contextual daily leisure time combined computer activities for girls only | | | |
| --- | --- | --- | --- | --- | --- | --- | --- | --- | --- | --- | --- | --- | --- | --- |
|  |  | β | p-value | 95% CI | β | p-value | 95% CI | β | p-value | 95% CI | β | p-value | 95% CI |  |
|  | Intercept | 0.442 | <0.001 | 0.245-0.640 | 0.501 | 0.003 | 0.166-0.836 | 0.432 | 0.011 | 0.098- 0.765 | 0.340 | 0.020 | 0.053-0.627 |  |
| Survey years | 2003 | Reference |  |  | Reference |  |  | Reference |  |  | Reference |  |  |  |
|  | 2007 | -0.050 | 0.010 | -0.088- -0.012 | -0.042 | 0.007 | -0.072- -0.012 | -0.038 | 0.048 | -0.077- -0.001 | -0.038 | 0.035 | -0.073- -0.003 |  |
|  | 2011 | -0.101 | <0.001 | -0.156- -0.046 | -0.085 | <0.001 | -0.127- -0.044 | -0.083 | 0.005 | -0.139 - -0.026 | -0.079 | 0.002 | -0.128- -0.030 |  |
|  | 2015 | -0.176 | <0.001 | -0.249- -0.104 | -0.156 | <0.001 | -0.215- -0.097 | -0.167 | <0.001 | -0.242 - -0.093 | -0.138 | <0.001 | -0.201- -0.076 |  |
| Individual-level variables | Between individual effects of daily computer activities | 0.053 | <0.001 | 0.042-0.064 | -0.005 | 0.345 | -0.015-0.005 | 0.031 | <0.001 | 0.017- 0.045 | 0.052 | <0.001 | 0.042-0.063 |  |
| Gender | Male | Reference |  |  | Reference |  |  |  |  |  |  |  |  |  |
|  | Female | -0.079 | <0.001 | -0.099- -0.059 | -0.073 | <0.001 | -0.092- -0.053 |  |  |  |  |  |  |  |
| Family affluence | Better off | Reference |  |  | Reference |  |  | Reference |  |  | Reference |  |  |  |
|  | About the same | -0.011 | 0.002 | -0.018- -0.004 | -0.012 | 0.002 | -0.019- -0.004 | -0.017 | <0.001 | -0.025 - -0.008 | -0.008 | 0.033 | -0.016- -0.001 |  |
|  | Less well off | 0.006 | 0.342 | -0.006-0.019 | 0.006 | 0.342 | -0.007-0.019 | -0.008 | 0.293 | -0.024- 0.007 | 0.016 | 0.009 | 0.004-0.028 |  |
| Leisure time activities | Less than once a week | Reference |  |  | Reference |  |  | Reference |  |  | Reference |  |  |  |
|  | Leisure time sports (Once a week) | -0.007 | 0.119 | -0.016-0.002 | -0.008 | 0.054 | -0.017- <0.000 | -0.004 | 0.454 | -0.015- 0.007 | -0.017 | 0.002 | -0.027- -0.006 |  |
|  | Leisure time reading books (Once a week) | -0.052 | <0.001 | -0.062- -0.042 | -0.054 | <0.001 | -0.063- -0.044 | -0.060 | <0.001 | -0.070 - -0.049 | -0.050 | <0.001 | -0.061- -0.039 |  |
|  | Leisure time going out (Once a week) | 0.261 | <0.001 | 0.232-0.291 | 0.263 | <0.001 | 0.233-0.293 | 0.273 | <0.001 | 0.244 - 0.302 | 0.250 | <0.001 | 0.217-0.282 |  |
|  | Leisure time hobbies (Once a week) | -0.022 | <0.001 | -0.029- -0.015 | -0.022 | <0.001 | -0.029- -0.015 | -0.011 | 0.002 | -0.017- -0.004 | -0.031 | <0.001 | -0.040--0.022 |  |
|  | Leisure time computer gaming (Once a week) | -0.016 | <0.001 | -0.025- -0.007 |  | NA |  |  | NA |  |  | NA |  |  |
|  | Leisure time internet use (Once a week) |  | NA |  | 0.053 | <0.001 | 0.041-0.065 |  | NA |  |  | NA |  |  |
| Contextual level variables | Between country differences in daily computer activities | -0.136 | 0.561 | -0.596-0.323 | -0.389 | 0.293 | -1.114-0.336 | -0.217 | 0.478 | -0.816- 0.382 | -0.316 | 0.255 | -0.861-0.229 |  |
|  | Change within countries from country mean in daily computer activities | 0.076 | 0.110 | -0.017-0.168 | -0.004 | 0.971 | -0.241-0.232 | 0.081 | 0.277 | -0.065- 0.227 | 0.007 | 0.900 | -0.103-0.117 |  |
|  | Between country differences in societal internet prevalence | 0.089 | 0.568 | -0.216-0.393 | -0.019 | 0.900 | -0.310-0.273 | 0.047 | 0.764 | -0.261- 0.356 | 0.209 | 0.142 | -0.070-0.487 |  |
|  | Change within countries from country mean in societal internet prevalence | -0.162 | 0.050 | -0.325-<0.0001 | -0.170 | 0.049 | -0.340- -0.001 | -0.116 | 0.261 | -0.318- 0.086 | -0.213 | 0.006 | -0.365--0.060 |  |
| Random effects | Country level variance | 0.012 |  | 0.008-0.019 | 0.012 |  | 0.007-0.018 | 0.014 |  | 0.009- 0.021 | 0.011 |  | 0.007- 0.017 |  |
|  | Country-year level variance | 0.002 |  | 0.001-0.002 | 0.002 |  | 0.001-0.002 | 0.002 |  | 0.001- 0.003 | 0.001 |  | 0.001- 0.002 |  |

Supplementary Table 5 – Results from two additional sensitivity analyses

|  |  | Model S5 heavy episodic drinking as an outcome of interest | | | Model S6 Without societal-level internet use | | |
| --- | --- | --- | --- | --- | --- | --- | --- |
|  |  | β | p-value | 95% CI | β | p-value | 95% CI |
|  | Intercept | 0.545 | <0.001 | 0.306-0.784 | 0.428 | 0.006 | 0.125- 0.731 |
|  | 2003 | Reference |  |  | Reference |  |  |
| Survey years | 2007 | -0.063 | 0.010 | -0.111- -0.015 | -0.018 | 0.290 | -0.050-0.015 |
|  | 2011 | -0.140 | <0.001 | -0.201- -0.079 | -0.047 | 0.061 | -0.096-0.002 |
|  | 2015 | -0.204 | <0.001 | -0.280- -0.127 | -0.107 | 0.001 | -0.168- -0.048 |
| Individual-level Variables | Between individual effects of daily computer activities | 0.038 | <0.001 | 0.027-0.049 | 0.043 | <0.001 | 0.033-0.054 |
| Gender | Male | Reference |  |  | Reference |  |  |
|  | Female | -0.067 | <0.001 | -0.090- -0.045 | -0.068 | <0.001 | -0.088- -0.048 |
| Family affluence | Better off | Reference |  |  | Reference |  |  |
|  | About the same | -0.015 | <0.001 | -0.022- -0.008 | -0.012 | 0.002 | -0.019- -0.004 |
|  | Less well off | 0.018 | 0.012 | 0.004-0.032 | 0.005 | 0.398 | -0.007-0.018 |
| Leisure time activities | Less than once a week | Reference |  |  | Reference |  |  |
|  | Leisure time sports (Once a week) | -0.018 | <0.001 | -0.027- -0.009 | -0.007 | 0.107 | -0.016-0.002 |
|  | Leisure time reading books (Once a week) | -0.082 | <0.001 | -0.097- -0.067 | -0.053 | <0.001 | -0.063- -0.043 |
|  | Leisure time going out (Once a week) | 0.282 | <0.001 | 0.257-0.306 | 0.263 | <0.001 | 0.233-0.293 |
|  | Leisure time hobbies (Once a week) | -0.040 | <0.001 | -0.048- -0.031 | -0.021 | <0.001 | -0.028- -0.014 |
| Contextual level variables | Between country differences in daily computer activities | -0.278 | 0.239 | -0.740-0.185 | -0.114 | 0.647 | -0.603-0.374 |
|  | Change within countries from country mean in daily computer activities | 0.124 | 0.124 | -0.034-0.283 | 0.087 | 0.148 | -0.031-0.205 |
|  | Between country differences in societal internet prevalence | 0.267 | 0.053 | -0.004-0.538 |  | NA |  |
|  | Change within countries from country mean in societal internet prevalence | -0.377 | <0.001 | -0.586- -0.169 |  | NA |  |
| Random effects | Country level variance | 0.008 |  | 0.005-0.014 | 0.012 |  | 0.008- 0.019 |
|  | Country-year level variance | 0.002 |  | 0.001-0.005 | 0.002 |  | 0.001-0.002 |
